# Associations Between Hearing Loss, Loneliness, and Social Isolation: A Systematic Review

**DOI:** 10.1093/geroni/igaa057.1018

**Published:** 2020-12-16

**Authors:** Jonathan Suen, Aishwarya Shukla, Adele Goman, Carrie Price, Frank Lin, Nicholas Reed

**Affiliations:** 1 Johns Hopkins University, Baltimore, Maryland, United States; 2 Johns Hopkins University School of Medicine, Baltimore, Maryland, United States

## Abstract

Hearing loss is highly prevalent among older adults, as is occurrences of loneliness and social isolation. Both loneliness and social isolation are also associated with insidious outcomes such as earlier mortality from all-causes and higher prevalence of chronic comorbidities. The purpose of this review is to synthesize published investigations that report on the associations between hearing loss with loneliness and social isolation. A systematic search through PubMed, Embase, CINAHL Plus, PsycINFO, and the Cochrane Library identified an initial total of 2495 references. Two independent reviewers screened articles for inclusion, with a third reviewer adjudicating. Studies published in English of older adults with hearing loss that also assessed loneliness and/or social isolation using a validated measure were included. Investigators used a modified Newcastle-Ottawa Scale (NOS) to appraise study quality. A final total of 14 articles were included in the review. The majority (12/14) were cross-sectional in design. Assessment methods were varied across hearing status, loneliness, and social isolation. Despite this heterogeneity, most multivariable adjusted investigations revealed that hearing loss was significantly associated with higher risks for both phenomena. Several studies also revealed this association to vary across gender, with women showing a stronger association than men. Our findings indicate that hearing loss is associated with both loneliness and social isolation, which have important implications for the cognitive and psychosocial health of older adults. Future investigations should examine possible underlying mechanisms of these relationships, as well as the efficacy of interventions through aural rehabilitation programs in addressing loneliness and social isolation.

